# MALDI-TOF MS for identification of *Tsukamurella* species: *Tsukamurella tyrosinosolvens* as the predominant species associated with ocular infections

**DOI:** 10.1038/s41426-018-0083-4

**Published:** 2018-05-09

**Authors:** Jade L. L. Teng, Ying Tang, Samson S. Y. Wong, Jordan Y. H. Fong, Zhe Zhao, Chun-Pong Wong, Jonathan H. K. Chen, Antonio H. Y. Ngan, Alan K. L. Wu, Kitty S. C. Fung, Tak-Lun Que, Susanna K. P. Lau, Patrick C. Y. Woo

**Affiliations:** 1 State Key Laboratory of Emerging Infectious Diseases, The University of Hong Kong, Hong Kong, China; 2 Research Centre of Infection and Immunology, The University of Hong Kong, Hong Kong, China; 3 Department of Microbiology, Li Ka Shing Faculty of Medicine, The University of Hong Kong, Hong Kong, China; 4 Carol Yu Centre for Infection, The University of Hong Kong, Hong Kong, China; 5 Department of Pathology, Pamela Youde Nethersole Eastern Hospital, Hong Kong, China; 6 Department of Pathology, United Christian Hospital, Hong Kong, China; 7 Department of Pathology, Tuen Mun Hospital, Hong Kong, China; 8 Collaborative Innovation Center for Diagnosis and Treatment of Infectious Diseases, The University of Hong Kong, Hong Kong, China

## Abstract

Although *Tsukamurella* infections have been increasingly reported in Europe, Asia, America, and Africa, indicating that diseases caused by this group of bacteria are emerging in a global scale, species identification within this genus is difficult in most clinical microbiology laboratories. Recently, we showed that *groEL* gene sequencing is useful for identification of all existing *Tsukamurella* species. Nevertheless, PCR sequencing is still considered expensive, time-consuming, and technically demanding, and therefore is yet to be incorporated as a routine identification method in clinical laboratories. Using *groEL* gene sequencing as the reference method, 60 *Tsukamurella* isolates were identified as five different *Tsukamurella* species [*T. tyrosinosolvens* (*n* = 31), *T. pulmonis* (*n* = 25), *T. hongkongensis* (*n* = 2), *T. strandjordii* (*n* = 1), and *T. sinensis* (*n* = 1)]. The most common source of the patient isolates were the eye (*n* = 18), sputum (*n* = 6), and blood (*n* = 6). None of the 60 isolates were identified correctly to species level by MALDI-TOF MS with the original Bruker database V.6.0.0.0. Using the Bruker database extended with 15 type and reference strains which covered all the currently recognized 11 *Tsukamurella* species, 59 of the 60 isolates were correctly identified to the species level with score ≥2.0. MALDI-TOF MS should be useful for routine species identification of *Tsukamurella* in clinical microbiology laboratories after optimization of the database. *T. tyrosinosolvens* was the most common species observed in patients with *Tsukamurella* infections and the predominant species associated with ocular infections.

## Introduction

The genus *Tsukamurella* contains clinically relevant species and cases have been increasingly reported in Europe, Asia, America, and Africa, indicating a global distribution of the bacteria^1–20^. The taxonomy of this genus has been continuously updated over the past few years. Recently, we have reclassified *Tsukamurella spongiae* DSM 44990, *Tsukamurella carboxydivorans* JCM 15482, and *Tsukamurella sunchonesis* JCM 15929 as later heterotypic synonyms of *Tsukamurella pulmonis*, *Tsukamurella tyrosinosolvens*, and *Tsukamurella pseudospumae*, respectively, based on the results of DNA–DNA hybridization and whole-genome sequencing^21, 22^. As a result, only 11 *Tsukamurella* species should be included in this genus according to the current state of the taxonomy at the time of writing^21, 22^. Among these 11 species, 8 are known to be associated with human infections, with the most common infections being indwelling device-related infections, meningitis, pulmonary, and cutaneous infections^23^. The disease spectra of *Tsukamurella* were further extended to ophthalmologic infections in recent years^1, 2, 24^. We have also discovered two novel *Tsukamurella* species, *Tsukamurella hongkongensis*, and *Tsukamurella sinensis*, from patients with keratitis and conjunctivitis respectively^25^. In addition to the association with various human infections, *Tsukamurella* can also be found in different environmental sources and animals^23, 26^^–^^29^. We have recently described another novel species, *Tsukamurella serpentis*, from the oral cavity of Chinese cobras^30^.

Accurate and rapid identification of bacteria is of critical importance in the diagnosis and management of infections. Although traditional phenotypic methods and commercial kits allow identification of most commonly encountered bacterial species in clinical microbiology laboratories, they often fail to differentiate *Tsukamurella* from the other related genera of the order *Corynebacteriales*^24, 31^, such as *Nocardia*, *Rhodococcus*, and *Gordonia*. Species identification within these genera is difficult in most clinical microbiology laboratories, as they share similar phenotypic properties. With the increasing availability of polymerase chain reaction (PCR) and DNA sequencing facilities, amplification, and sequencing of universal gene targets represents an advanced technology that theoretically yields unambiguous identification results, especially in cases where bacterial isolates are difficult to identify by phenotypic tests. Among the various studied gene targets, the 16S ribosomal RNA (rRNA) gene has been the most widely used for bacterial identification and classification^32, 33^. However, our previous study showed that this gene target cannot be confidently used for species identification of *Tsukamurella* as highly similar 16S rRNA gene sequence can be shared by different *Tsukamurella* species^34^. Sequencing of an alternative gene target, the *groEL* gene, was shown to be useful and accurate for identification of all existing *Tsukamurella* species^34^. Nevertheless, PCR sequencing is still considered expensive, time-consuming and technically demanding, and therefore is yet to be incorporated as a routine identification method in clinical microbiology laboratories.

Matrix-assisted laser desorption ionization time-of-flight mass spectrometry (MALDI-TOF MS) has recently emerged as a revolutionary technique for identification of bacterial and fungal pathogens, yielding rapid, accurate and highly reproducible results at a lower price than any other methods routinely used in clinical microbiology laboratories^35^^–^^39^. The application of MALDI-TOF MS for identification of *Tsukamurella* species has not been fully explored to date. So far, there were only two studies that have reported on the evaluation of MALDI-TOF MS for identification of *Tsukamurella*, with only two *Tsukamurella* strains being included in each series^40, 41^. In this study, using *groEL* gene sequencing as the reference method, we evaluated the performance of MALDI-TOF MS using the Bruker Biotyper system for identification of 60 clinical *Tsukamurella* isolates.

## Results

### *groEL* gene sequence analysis of *Tsukamurella* isolates

**Fig. 1:**
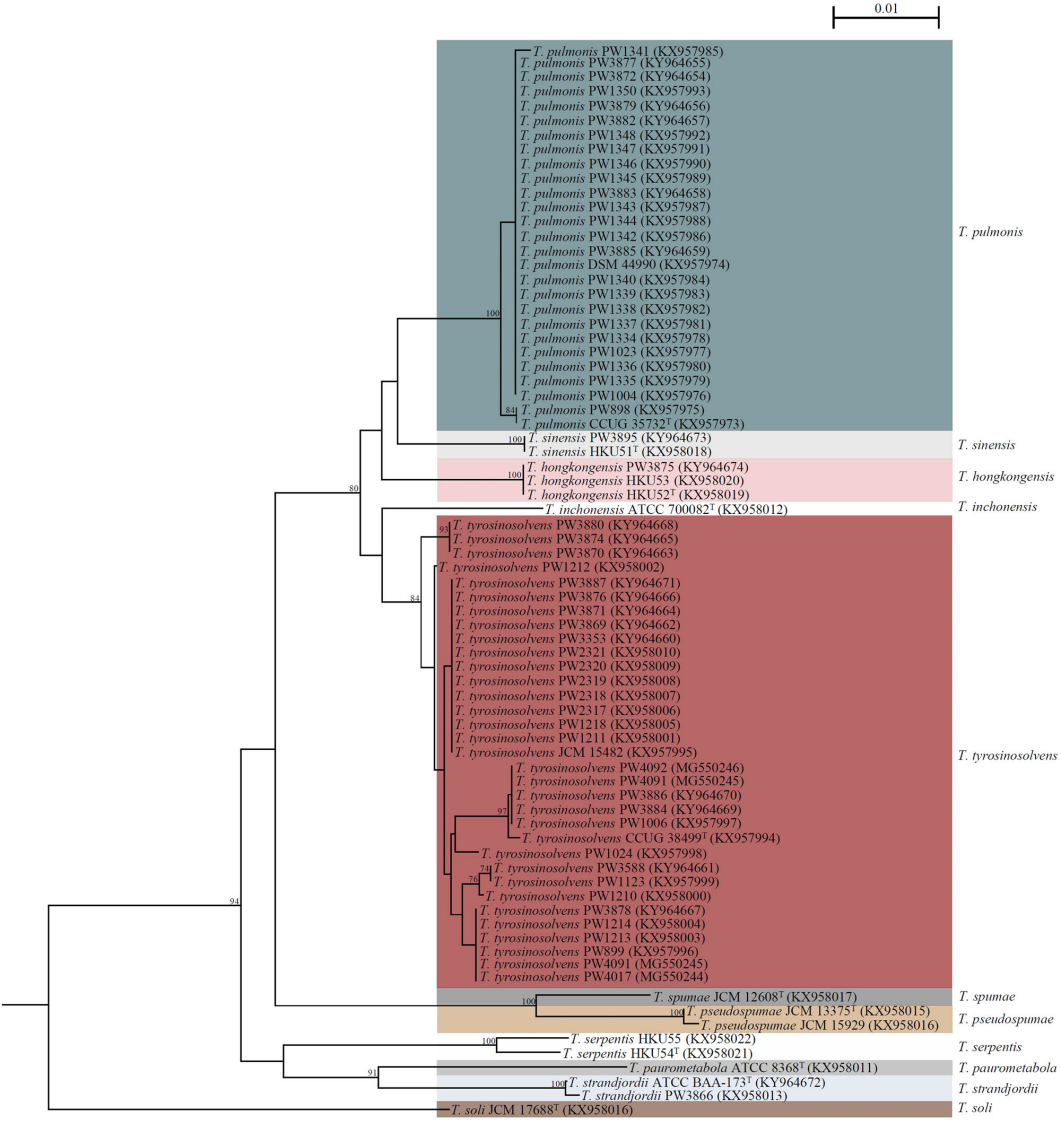
Phylogenetic trees showing the relationship of *Tsukamurella* strains included in this study, including 15 type and reference strains and 60 *Tsukamurella* isolates. The tree was inferred from partial *groEL* sequence data (677 nucleotide positions of the trimmed sequence alignments respectively) by the maximum-likelihood method using the model GTR + I + G and *Mycobacterium smegmatis* MC^2^ 155 (CP009494.1) as the outgroup. The scale bar indicates the estimated number of substitutions per base. Numbers at nodes indicate levels of bootstrap support calculated from 1000 trees and expressed as percentage. All names and accession numbers are given as cited in the GenBank database

Using *groEL* gene sequencing as the reference method, the 60 *Tsukamurella* isolates included in this study were identified as five different *Tsukamurella* species, including *T. tyrosinosolvens* (*n* = 31, 51.7%), *T. pulmonis* (*n* = 25, 41.7%), *T. hongkongensis* (*n* = 2, 3.3%), *T. strandjordii* (*n* = 1, 1.7%), and *T. sinensis* (*n* = 1, 1.7%). Among the 25 *T. pulmonis* strains identified, 16 (64.0%) were isolated from the oral cavities of Chinese cobras while the remaining 9 (36.0%) were isolated from patients. *groEL* gene sequence analysis showed that intraspecies nucleotide identities among the 27 strains of *T. tyrosinosolvens*, the 25 strains of *T. pulmonis* and the two strains of *T. hongkongensis* ranged from 98.7 to 100.0%, 99.7 to 100.0% and 100.0%, respectively. Phylogenetic tree construction based on the *groEL* gene sequences revealed that all the 60 isolates clustered correctly with their corresponding type and reference strains of the same species (Fig. 1).

### Clinical spectra of *Tsukamurella* infections in Hong Kong

Including the clinical isolates that we previously reported in our locality^1, 2, 34^, the most common *Tsukamurella* species observed in our patients was *T. tyrosinosolvens* (*n* = 31), followed by *T. pulmonis* (*n* = 9), *T. hongkongensis* (*n* = 3), *T. sinensis* (*n* = 2), and *T. strandjordii* (*n* = 1). These *Tsukamurella* isolates were recovered from different types of clinical specimens, most commonly eye (*n* = 18), sputum (*n* = 6), and blood (*n* = 6) (Table 1).

**Table 1:** Summary of *Tsukamurella* species isolated from different clinical specimens

Type of specimen	*T. tyrosinosolvens* (*n* = 31)	*T. pulmonis* (*n* = 9)	*T. hongkongensis* (*n* = 3)	*T. sinensis* (*n* = 2)	*T. strandjordii* (*n* = 1)
Eye (*n* = 18)	11	4	1	2	0
Sputum (*n* = 6)	4	0	1	0	1
Blood (*n* = 6)	4	1	1	0	0
Others (*n* = 9)					
Axillary lymph node biopsy^a^	1	0	0	0	0
Plantar granulation tissue^a^	2	0	0	0	0
Lung tissue^a^	1	0	0	0	0
Peritoneal tissue^a^	1	0	0	0	0
Peritoneal dialysis fluid	1	0	0	0	0
Pus swab from submandibular skin	0	1	0	0	0
Superficial wound swab from hand	1	1	0	0	0
Unknown specimens (*n* = 7)	5	2	0	0	0

^a^ The recovery of these isolates was due to laboratory contamination during processing of the tissue specimens [previously reported in ref. 29]

### Species identification of *Tsukamurella* by MALDI-TOF MS

**Fig. 2:**
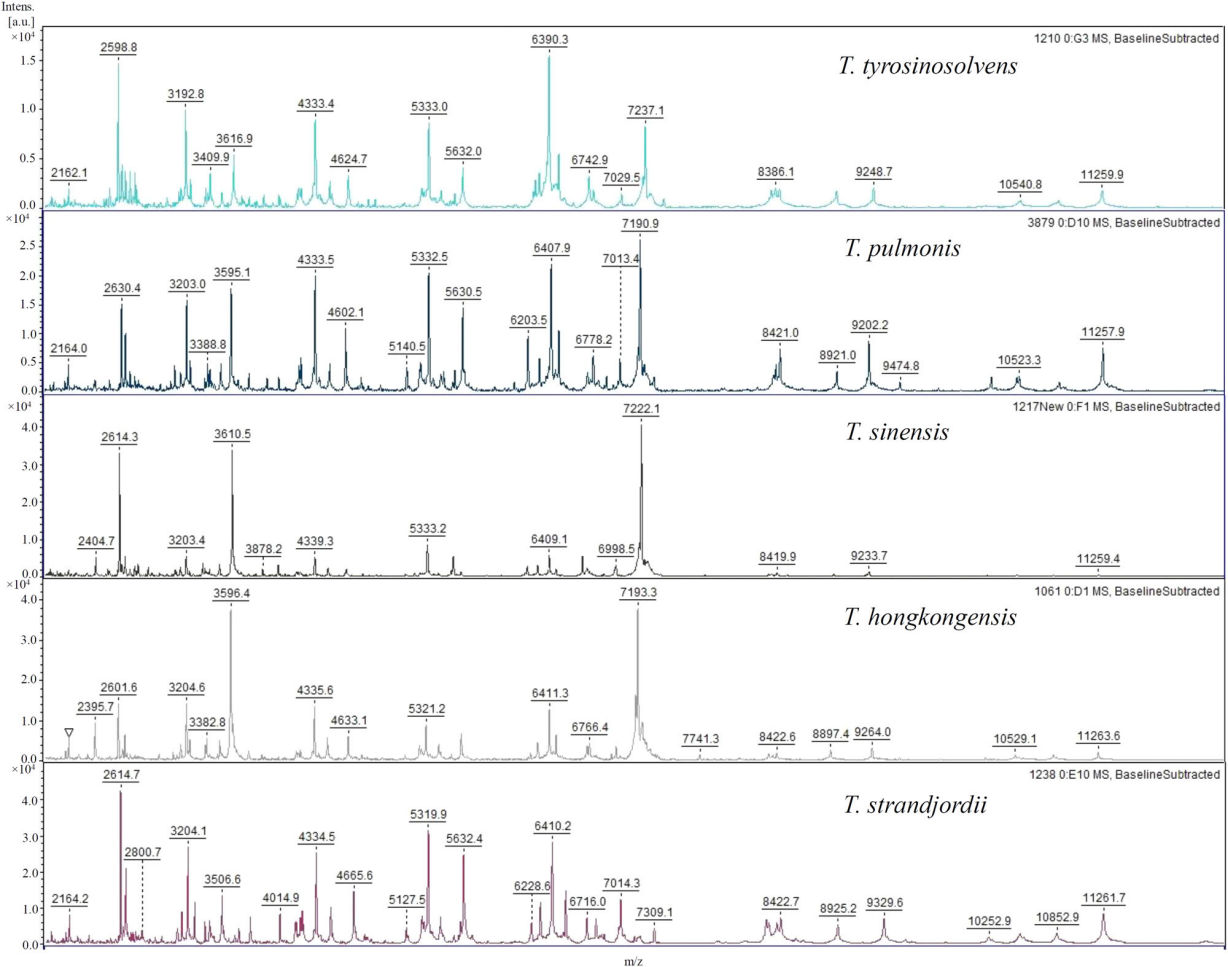
MALDI-TOF MS spectra of *T. tyrosinosolvens*, *T. pulmonis*, *T. hongkongensis*, *T. strandjordii*, and *T. sinensis*. The intensity in arbitrary units [a.u.] are shown on the *y* axis, and the masses (*m*/*z*) of the ions are shown on the *x* axis. The *m*/*z* values represent the mass-to-charge ratios

The MALDI-TOF MS results of the 15 *Tsukamurella* type and reference strains using the Bruker reference library V.6.0.0.0 (6903 spectra) were shown in Table 2. Using *groEL* gene sequencing as the reference method for species identity, 14 (93.3%) of the 15 isolates were identified correctly to the genus level but only 6 (40.0%) showed the correct genus with score ≥1.7. One isolate showed incorrect genus identification. *T. soli* was misidentified as *Corynebacterium jeikeium* with score <1.7 (Table 2). Of the 14 isolates identified to the genus level, only 2 (14.3%) showed the correct species with score ≥2.0 (Table 2). Of the 13 strains which showed incorrect species identification, all were due to the absence of the corresponding species in the database. Therefore, reference spectra which covered all the currently recognized 11 *Tsukamurella* species were included in the reference library. The spectra of some commonly observed *Tsukamurella* species, including *T. tyrosinosolvens*, *T. pulmonis*, *T. hongkongensis*, *T. strandjordii*, and *T. sinensis*, were shown in Fig. 2. Using the extended in-house Bruker database, all the 15 type and reference strains were identified correctly to the species level with score ≥2.0 (Table 2).

**Table 2:** MALDI-TOF MS results of 75 *Tsukamurella* species and 14 species of closely related genera using Bruker database and extended in-house database

Bacterial species	Total no. of isolates (*n* = 89)	No. of isolates resulting in the indicated score attained using:							
		Bruker Reference Library V.6.0.0.0				Extended in-house database			
		≥2.0	1.7–1.9	<1.7	Incorrect species identification	≥2.0	1.7–1.9	<1.7	Incorrect species identification
Type and reference (*n* = 15)^a^									
* T. paurometabola*	1	1	0	0	0	1	0	0	0
* T. inchonensis*	1	1	0	0	0	1	0	0	0
* T. strandjordii*	1	0	0	1	1	1	0	0	0
* T. pulmonis*	2	0	1	1	2	2	0	0	0
* T. tyrosinosolvens*	2	2	0	0	2	2	0	0	0
* T. pseudospumae*	2	0	0	2	2	2	0	0	0
* T. spumae*	1	0	1	0	1	1	0	0	0
* T. soli*	1	0	0	1	1	1	0	0	0
* T. serpentis*	1	0	0	1	1	1	0	0	0
* T. hongkongensis*	1	0	0	1	1	1	0	0	0
* T. serpentis*	2	0	0	2	2	2	0	0	0
Clinical or veterinary (*n* = 74)									
* T. tyrosinosolvens*	31	20	10	1	31	31	0	0	0
* T. pulmonis*	25	0	9	16	25	25	0	0	0
* T. hongkongensis*	2	0	0	2	2	1	1	0	1
* T. sinensis*	1	0	0	1	1	1	0	0	0
* T. strandjordii*	1	0	0	1	1	1	0	0	0
* N. nova*	3	3	0	0	0	3	0	0	0
* N. cyriacigeorgica*	2	1	1	0	0	1	1	0	0
* N. brasiliensis*	1	0	1	0	0	0	1	0	0
* N. farcinica*	2	1	1	0	0	1	1	0	0
* R. equi*	2	2	0	0	0	2	0	0	0
* R. erythropolis*	1	1	0	0	0	1	0	0	0
* G. sputi*	2	2	0	0	0	2	0	0	0
* G. bronchialis*	1	1	0	0	0	1	0	0	0

^a^ Among the 15 type and reference strains, only 6 (40.0%) showed the correct genus with score ≥1.7 by MALDI-TOF MS using the Bruker Reference Library V.6.0.0.0

The MALDI-TOF MS results of the 60 *Tsukamurella* isolates, including patient (*n* = 44) and veterinary (*n* = 16) isolates, using the Bruker reference library V.6.0.0.0 (6903 spectra), was shown in Table 2. Using *groEL* gene sequencing as the reference method for species identification, all isolates were identified correctly to the genus level but only 39 (65.0%) showed the correct genus with score ≥1.7, and none of the 60 isolates were identified correctly to the species level (Table 2). Using the extended in-house Bruker database, 59 (98.3%) of the 60 isolates were correctly identified to the species level with score ≥2.0 [54 (90.0%) with score of top match ≥2.0 and score of second match lower by ≥10%]. One of the two strains of *T. hongkongensis* was misidentified as *T. inchonensis* (score <2.0) though the MSP of *T. hongkongensis* has been already included in the extended in-house database.

Using both the Bruker reference library V.6.0.0.0 and the extended in-house database, all the 14 clinical isolates belonging to genera that are closely related to *Tsukamurella*, including *Nocardia* [*N. nova* (*n* = 3); *N. cyriacigeorgica* (*n* = 2); *N. brasiliensis* (*n* = 1); *N. farcinica* (*n* = 2)], *Rhodococcus* [*R. equi* (*n* = 2); *R. erythropolis* (*n* = 1)], and *Gordonia* [*G. sputi* (*n* = 2); *G. bronchialis* (*n* = 1)], were identified correctly to species level. Among these, 11 (78.6%) showed correct species with score ≥2.0 (Table 2).

### Clustering analysis of the spectra of *Tsukamurella* generated by the bruker biotyper

**Fig. 3:**
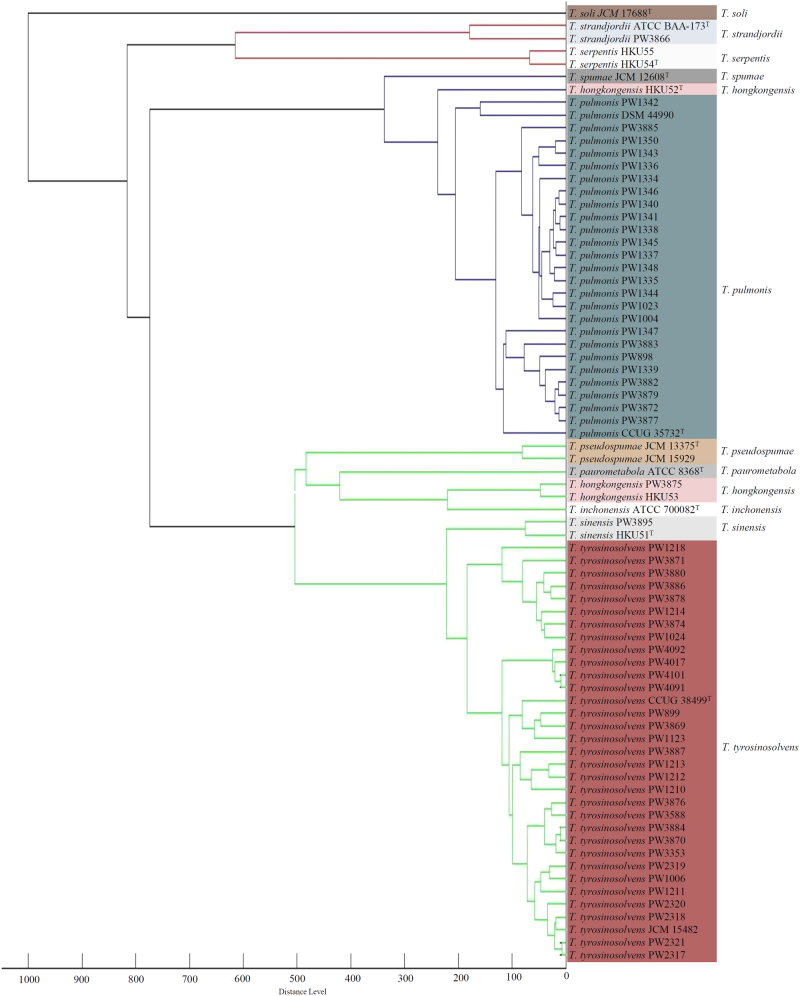
Dendrogram generated from hierarchical cluster analysis of MALDI-TOF MS spectra of 15 type and reference strains and 60 *Tsukamurella* isolates included in this study. Distances are displayed in relative units

Dendrogram generated from hierarchical cluster analysis of MALDI-TOF MS showed that the spectra of the 15 *Tsukamurella* type and reference strains and 60 *Tsukamurella* isolates were able to form distinct clusters for each *Tsukamurella* species, except for one strain of *T. hongkongensis*, which was not able to cluster with the other two strains of *T. hongkongensis* (Fig. 3). Except this particular *T. hongkongensis* strain, the topology of the dendrogram was completely concordant to that of the phylogenetic tree constructed based on *groEL* gene sequence (Fig. 1).

### GenBank nucleotide sequence accession numbers

The nucleotide sequences of the partial *groEL* gene sequences of *Tsukamurella* isolates obtained in the present study have been lodged within the Genbank sequence database under accession numbers KY964654-KY964674 and MG550244-MG550247.

## Discussion

MALDI-TOF MS should be useful for routine species identification of *Tsukamurella* in clinical microbiology laboratories after optimization of the database by adding reference MSPs of all the known *Tsukamurella* species. Differentiation of *Tsukamurella* from other related genera of the order *Corynebacteriales*, such as *Nocardia*, *Rhodococcus*, and *Gordonia*; and species identification within these genera are difficult in most clinical microbiology laboratories, as they share similar phenotypic properties. Recently, we showed that *groEL* gene sequencing is a reliable molecular diagnostic method for species identification of *Tsukamurella*^34^. However, identification by sequencing is still beyond the reach of many routine clinical laboratories. In contrast, MALDI-TOF MS is user-friendly, rapid, and cost-effective. Most importantly, this method allows the addition of MSPs of bacterial species not included in the database to improve its performance for bacterial species identification. In this study, all the 60 isolates were identified correctly to the genus level using the Bruker database V.6.0.0.0 (6903 spectra). However, all of them were misidentified at the species level, which is in line with the results of the two previous studies where none of the four *Tsukamurella* strains tested, including two strains of *T. tyrosinosolvens* and two strains of unknown species identity, could be identified correctly to the species level^40, 41^. This is most likely due to the absence of a comprehensive *Tsukamurella* spectral database in the Bruker system which, at the time of writing, includes only the spectra of two species, *T. paurometabola* and *T. inchonensis*. In view of this problem, we optimized the database by adding 15 MSPs covering all the known *Tsukamurella* species. Using the expanded in-house database, 59 (98.3%) of the 60 *Tsukamurella* isolates can be correctly identified to species level with score ≥2.0. This marked increase in accuracy after adding MSPs in the database is in line with results of our previous study, which also showed that by including 21 *B. pseudomallei* MSPs in the Bruker database, all 31 clinical, veterinary and environmental isolates of *B. pseudomallei* were correctly identified^42^. In addition to the improved identification accuracy, such in-house database can also facilitate the rapid species identification of *Tsukamurella*. We previously reported a study of *Tsukamurella* pseudo-outbreak due to environmental contamination of laboratory device^29^. The species identities of the concerned *Tsukamurella* isolates (*n* = 5) were determined as *T. tyrosinosolvens* after 10 days of extensive diagnostic workup^29^. In contrast, using the expanded in-house Bruker database developed in this study, all the concerned isolates could be rapidly identified as *T. tyrosinosolvens* within an hour, highlighting this technology could markedly shorten the turnaround time for identification of *Tsukamurella*.

The number of strains for each species in MALDI-TOF MS databases should also be expanded to cover intraspecies variability to improve the performance and accuracy for species identification. Although we have added one reference spectrum of *T. hongkongensis* in the MALDI-TOF MS database, not all the strains of *T. hongkongensis* could be correctly identified. There were two test strains of *T. hongkongensis* included in the present study, one showed correct species identification with high score (score ≥2.0), whereas the other test strain was misidentified as *T. inchonensis* with score <2.0 (Table 2). Consistently, dendrogram also revealed that one *T. hongkongensis* strain was separated from the *T. hongkongensis* cluster, being more related to the *T. pulmonis* cluster (Fig. 3). We speculate that the wrong identification or clustering may be due to the inclusion of just one *T. hongkongensis* MSP in the database. We anticipate that correct identification of this test strain can be achieved when more MSPs belonging to the species of *T. hongkongensis* is included. This is also in line with our previous observation that our three *Burkholderia thailandensis* isolates were originally misidentified as *B. pseudomallei* when the database contained only one *B. thailandensis* MSP, but expansion of the database with one additional *B. thailandensis* reference strain enabled correct identification of two other *B. thailandensis* isolates^42^. Increasing the number of available spectra for each species is essential to cover intraspecies variability, thereby improving the accuracy of MALDI-TOF MS for identification of *Tsukamurella*.

*T. tyrosinosolvens* was the most common species observed in patients with *Tsukamurella* infections, especially ocular infections, in our locality. Among the 46 patient isolates included in the present study, 31 (67.4%) were identified as *T. tyrosinosolvens*, followed by *T. pulmonis* (*n* = 9, 19.6%). The predominance of *T. tyrosinosolvens* is similar to that of Taiwan where *T. tyrosinosolvens* was also the most commonly encountered *Tsukamurella* species in their population^24^. Among the cases reported in the literature during 1992–2016, *T. tyrosinosolvens* also appeared to be more prevalent than other *Tsukamurella* species^1–14, 18–20^. However, most of these studies were relied on phenotypic tests and/or 16S rRNA gene sequencing for species identification, which may not offer sufficient discriminative power for species discrimination^3, 5–8, 10, 13, 14, 20^. Similar studies in other countries using *groEL* gene sequencing or MALDI-TOF MS with an expanded in-house Bruker database for species identification of *Tsukamurella* are required to more accurately assess their relative clinical importance and ascertain the emergence and pathogenic potential of *T. tyrosinosolvens*.

## Materials and methods

### Type and reference strains

A total of 15 type and reference strains of the genus *Tsukamurella*, representing 11 species, were obtained from four public culture collections, with *T. pulmonis* CCUG 35732^T^ and *T. tyrosinosolvens* CCUG 38499^T^ obtained from Culture Collection, University of Göteborg, *T. paurometabola* ATCC 8368^T^, *T. strandjordii* ATCC BAA-173^T^, and *T. inchonensis* ATCC 700082^T^ from American Type Culture Collection, *T. pulmonis* DSM 44990 from Leibniz Institute DSMZ – German Collection of Microorganisms and Cell Cultures and *T. hongkongensis* JCM 30715^T^, *T. sinensis* JCM 30714^T^, *T. serpentis* JCM 31017^T^, *T. serpentis* JCM 31018, *T. soli* JCM 17688^T^, *T. pseudospumae* JCM 15929, *T. pseudospumae* JCM 13375^T^, *T. spumae* JCM 12608^T^, and *T. tyrosinosolvens* JCM 15482 from Japan Collection of Microorganisms.

### Patient and veterinary isolates

In addition to the 15 *Tsukamurella* type and reference strains, 60 *Tsukamurella* clinical or veterinary isolates were included in this study. Among these, 44 were isolated from patients whereas 16 were isolated from the oral cavities of healthy Chinese cobras (*Naja atra*). Of the 44 human isolates, they were recovered from different types of specimens from in-patients of four different hospitals during 2004 to 2017. The presumptive identification of all the 60 isolates to the genus level was initially made by conventional biochemical methods (i.e., displayed as typical dry and rough colonies on horse blood agar, appeared as Gram-positive rods, and negative for Ziehl–Neelsen stain but positive for modified Ziehl-Neelsen stain) prior to performing *groEL* gene sequencing for confirmation of their species identities^34, 43^. The species identities of the 34 isolates were confirmed in a previous study^34^, whereas those of the remaining 26 isolates were confirmed in the present study by *groEL* gene sequencing using the validated threshold value of 98.2% for species identification^34^. Fourteen clinical isolates belonging to genera that are closely related to *Tsukamurella*, including *Nocardia* [*N. nova* (*n* = 3); *N. cyriacigeorgica* (*n* = 2); *N. brasiliensis* (*n* = 1); *N. farcinica* (*n* = 2)], *Rhodococcus* [*R. equi* (*n* = 2); *R. erythropolis* (*n* = 1)], and *Gordonia* [*G. sputi* (*n* = 2); *G. bronchialis* (*n* = 1)] were used to evaluate the extended in-house database.

### DNA extraction

Bacterial DNA extraction was modified from our previous published protocol^25^. Briefly, bacterial cells were harvested and suspended in sterile distilled water. One microliter of RNase A (10 mg/mL) (QIAGEN, Hilden, Germany) and 2 μL of lysozyme (100 mg/mL) (Sigma Aldrich, St Louis, MO, USA) were added into the cell suspension and the mixture was incubated at 37 °C for 60 min to break down the bacterial cell wall. Two microliters of proteinase K (600 mAU/mL) (QIAGEN) and 120 μL of SDS (10%) (Sigma Aldrich) were then added into the mixture and incubated at 65 °C for 60 min to lyse the cells. Subsequently, 420 μL of ammonium acetate (8 M) (Sigma Aldrich) was added into the cell lysate and the mixture was chilled on ice immediately for 5 min followed by centrifuged at 16,100 × *g* for 20 min. The supernatant was collected, mixed with an equal volume of isopropanol to precipitate DNA overnight at 4 °C. On the following day, the cell pellet was washed with 70% ethanol and DNA was re-dissolved in 25 μL of sterile distilled water. The concentration was measured by a micro-volume UV-visible light spectrophotometer (NanoDrop 2000) (Thermo Scientific, Waltham, MA, USA). The resultant mixture was diluted 100× and 0.5 μL of the diluted extract was used for PCR.

### PCR amplification and sequencing

Extracted DNA from the 25 isolates of *Tsukamurella* was used as the template for amplification of *groEL* gene using primers LPW34162 (5′-GAC GCT CAT CGT CAA CAA GAT CC-3′) and LPW33894 (5′-GGA CTY AGA AGT CCA TGC CGC CCA T-3′). The PCR mixture (25 μL) contained denatured *Tsukamurella* genomic DNA, 4% of DMSO (Bio-Rad, Hercules, CA, USA), PCR buffer (10 mM Tris-HCl pH 8.3 and 50 mM KCl), 2 mM MgCl_2_, 10 μM forward and reverse primers, 200 μM of each deoxynucleoside triphosphates and 2.5 U Ampli*Taq* Gold DNA polymerase (Applied Biosystems, Foster City, CA, USA). The sample was amplified in 40 cycles of 94 °C for 1 min, 55 °C for 1.5 min and 72 °C for 2 min, and with a final extension at 72 °C for 7 min in an automated thermal cycler (Applied Biosystems). Five microliters of each amplified product was electrophoresed in 2% (w/v) agarose gel, with a molecular size marker (GeneRuler™ 50 bp DNA ladder, MBI Fermentas, Canada). Electrophoresis in Tris-borate-EDTA buffer was performed at 100 volts for 45 min. The gel was stained with ethidium bromide (0.5 μg/mL) for 20 min, rinsed and photographed under ultraviolet light illumination.

The PCR product was gel-purified using the QIAquick PCR purification kit (QIAGEN). Both strands of the PCR product were sequenced using BigDye Terminator Cycle Sequencing kit version 3.1 with an ABI Prism 3730XL Analyzer according to manufacturer’s instructions (Applied Biosystems) and the PCR primers of respective gene targets.

### Phylogenetic analyses

The sequences of the PCR products were compared with sequences of all currently recognized *Tsukamurella* species by multiple sequence alignment using MUSCLE 3.8^44^ and the aligned sequences were trimmed using BioEdit 7.2.0^45^. Comparative gene sequence analysis was performed using BioEdit 7.2.0. Phylogenetic tree was constructed by maximum-likelihood method using MEGA version 6^46^.

### MALDI-TOF MS analysis

Sample preparation for MALDI-TOF MS analysis using Bruker Biotyper was performed as previously described with modifications^47^. Briefly, bacterial strains were grown on blood agar at 37 °C for 48 h. Two to three colonies were harvested in 500 μL of sterile water and boiled for 30 min followed by centrifugation at 13,000 × *g* for 2 min to remove the supernatant. Sterile water (300 μL) and pure ethanol (900 μL) were added to the suspension and mixed. The suspensions were centrifuged twice at 13,000 × *g* for 2 min to remove the supernatant. The pellets were dried at room temperature and suspended in 50 μL of 70% formic acid (Sigma-Aldrich) and 50 μL of acetonitrile (Sigma-Aldrich). After centrifugation at 13,000 × *g* for 2 min, 1 μL of supernatant was spotted on a polished steel target plate. Immediately after drying, spots were overlaid with 1 μL of matrix solution. Samples were processed in Bruker Biotyper (Bruker Daltonics, GmbH, Bremen, Germany) system. Spectra within the range of *m*/*z* 2000–20,000 Da were obtained with an accelerating voltage of 20 kV in linear mode and analyzed with MALDI Biotyper 3.1 and Reference Library V.6.0.0.0 (6903 spectra). Bruker bacterial test standard (BTS, no. 255343, Bruker Daltonics) was used for calibration and quality control in each run. Thresholds for species and genus identification were ≥2.0 and ≥1.7, respectively. Results were presented with the species and genus identification and compared to identification results by *groEL* gene sequencing. Scores below the cutoff were considered invalid results with the conclusion “not reliable identification”. Furthermore, the mass spectrum profile (MSP) created from spectra of each strain was used for hierarchical cluster analysis using the Bruker Biotyper software with default parameters, where distance values were relative and normalized to a maximum value of 1000^48^.

### Establishment of a *Tsukamurella* database

The procedures for establishing an in-house *Tsukamurella* database was performed as previously described with modifications^47^. One microliter of the final extraction product of the *Tsukamurella* strain was spotted four times onto a steel target and each spot was measured six times for collecting a total of 24 individual, high-quality spectra per isolate. The spectra were then analyzed in the flexAnalysis software (version 3.0, Bruker Daltonics) to generate a single mean spectrum for each *Tsukamurella* reference strain using the Biotyper MSP creation standard method as described previously^49^.
